# The safety of biocleaning technologies for cultural heritage

**DOI:** 10.3389/fmicb.2014.00155

**Published:** 2014-04-10

**Authors:** Pilar Bosch-Roig, Giancarlo Ranalli

**Affiliations:** ^1^Department of Conservation and Restoration of Cultural Heritage, Instituto Universitario de Restauración del Patrimonio, Universitat Politècnica de ValènciaValencia, Spain; ^2^Laboratorio di Microbiologia Ambientale e Biorestauro, Dipartimento di Bioscienze e Territorio, Università del MolisePesche, Italy

**Keywords:** safety, biocleaning, biotechnology, microorganisms, cultural heritage

## Microbial biodiversity and ecology for biocleaning technologies

It is well known that in nature microorganisms play important ecological roles both in the food chain and in biogeochemical cycles, such as in the nitrogen, carbon, phosphorus, and sulphur cycles, for example. Microorganisms display wide diversity in enzyme production, including lipases, proteases, and oxido-reductases, as described by metagenomic studies (Neelakanta and Sultana, 2013). Microorganisms are ubiquitous, having been able to thrive and survive in every part of the biosphere, in the soil, on rocks, in hot springs, in the oceans, in the atmosphere and so on, because of their great adaptability to environmental conditions. Microorganism biodiversity includes Bacteria, Archaea, and Eukaryotes (Woese, 2000), which are extraordinarily diverse in their requirements for growth and their proliferation is greatly affected by the nutrients that are available in their environment. However, they have common living requirements: energy, from light or from organic or inorganic compounds; macronutrients including carbon, nitrogen, and hydrogen; trace elements such as Co, Zn, Cu, and Mn and water. There are, therefore, some common environmental factors that influence their growth: the availability of water and oxygen, temperature, salinity, and pH (Caneva et al., 2008).

The productive use and exploitation of microorganisms by our society has ancient origins and there are widespread applications in food production, health-care, energy-production, wastewater treatment, and agriculture.

Research in the field of cultural heritage biological cleaning started in the beginning of 1990s and have since then artwork applications has highly increased. Today it is recognized as being a viable alternative to traditional chemical treatments such as organic solvents or other aggressive conservation methods like mechanical treatments. Microorganisms are the new bioagents for the recovery and conservation of artwork and historical architectural monuments. The basic idea of these innovative biological methods (biocleaning, bioconsolidation) is encouraged by the fact that only a few known microorganisms play a destructive role (causing deterioration) in the natural processes, while the majority of them are responsible for “virtuous” processes. In addition, microorganisms can have advantages over chemical methods and enzymes in cleaning Cultural Heritage (CH), especially when the substances to be cleaned are complex and encrusted, due to their specificity of a pool of enzyme production (Ranalli and Sorlini, 2008). Careful selection of the appropriate (not pathogenic) microorganisms with the requisite characteristics for the removal of undesirable substances (nitrates, sulfates, organic matter, etc.) is one of the first steps to be taken in formulating the best bio-restoration strategy. Microorganisms can be bought from international collections of microorganisms (ATCC, CBS, DSMZ, etc.) or isolated from environmental matrices. Soil is one of the most abundant sources of microorganisms with up to 4 × 10^6^ taxa/ton of soil (Curtis et al., 2002). Due to the great biodiversity in existing microorganisms, the selection of natural microorganisms using microbiological techniques such as culture dependent enrichment and/or culture independent molecular methods permits the isolation of the appropriate microorganisms for biological processes for CH purposes without resorting to the use Genetically Modified Organism (GMO), which could potentially, through diffusion of the biological techniques lead to additional, unforeseen risks to safety.

## Biocleaning technologies overview

**Figure 1:**
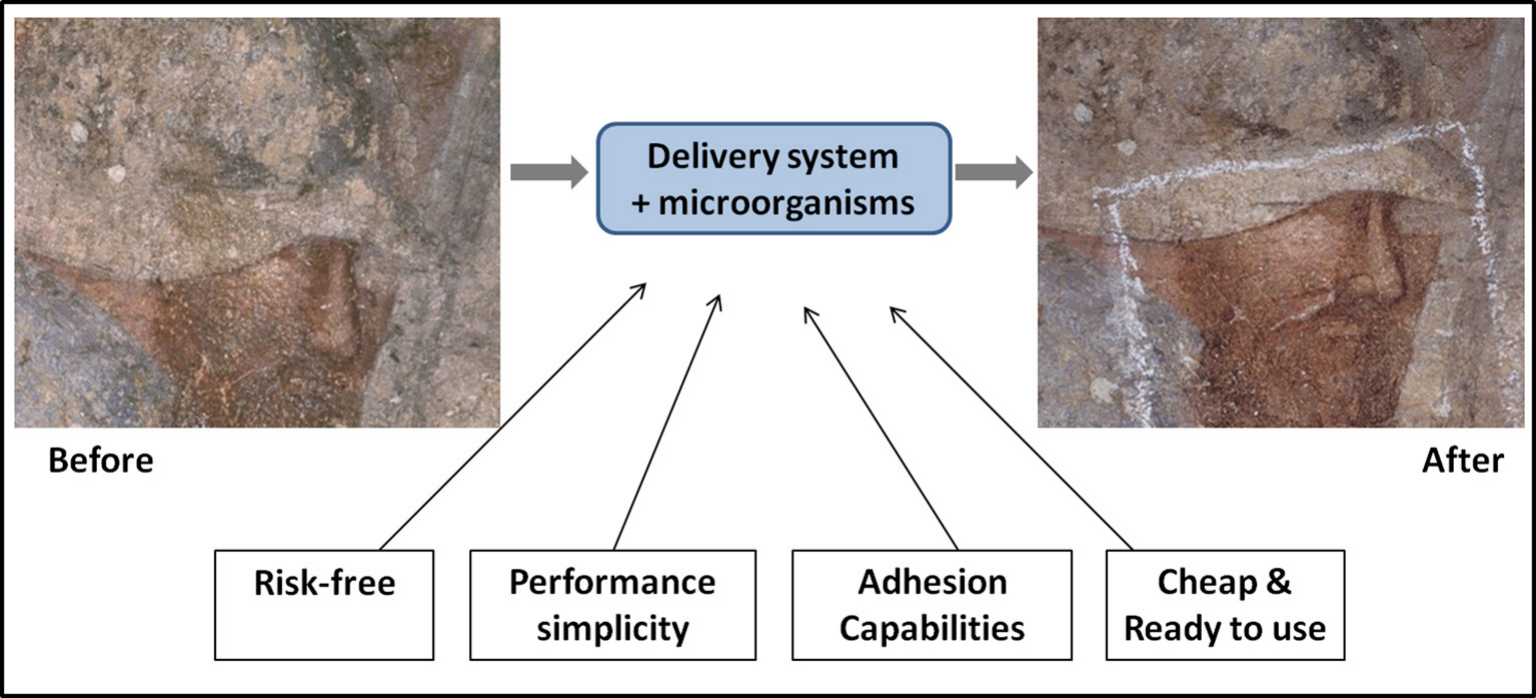
**Main characteristics and effects of biocleaning process (bacteria and enzyme) on a fragment on Conversione di S. Efisio e battaglia, fresco (XIV century) at Pisa Camposanto Monumentale, (Italy)**.

Biocleaning technologies applied to CH have evolved to function in a wide range of environments, from laboratory conditions to Cultural Heritage monuments like the Camposanto Monumentale cemetary in Pisa, Italy (Figure 1); the Santos Juanes Church in Valencia, Spain; the Duomo di Milano Cathedral in Milan, Italy; Matera Cathedral, Matera, Italy; the Duomo di Firenze Cathedral in Florence, Italy; a nineteenth-century building in Riga, Latvia; the Epidauro Theatre in Greece and artworks like Michelangelo's “Pietà Rondanini” and sculpture by Lina Arpesani. Bio-technologies have been able to resolve a range of problems on various artistic materials (including monumental stone, wall paintings, marble statues, etc.) and to combat diverse artistic pathologies (such as the bioremoval of organic substances, black crusts, and mineral salts) by using different cultures of viable bacteria. Sulphate-reducing bacteria like *Desulfovibrio desulfuricans* and *Desulfovibrio vulgaris;* nitrate-reducing bacteria like *Pseudomonas stutzeri*, and others. (Heselmeyer et al., 1991; Gauri et al., 1992; Ranalli et al., 2000, 2003, 2005; Cappitelli et al., 2005, 2007; Polo et al., 2010; Alfano et al., 2011; Gioventù et al., 2011; Bosch-Roig et al., 2012, 2013a,b; Troiano et al., 2013).

## Safe biocleaning

Intense research on advanced microbiological systems based on the use of microorganisms for the removal of alterations on works of art have shown them to be a viable alternative for CH restoration. Various regional and national projects and one European project (BIOBRUSH EVK4-2001-00055) have provided economic support for research into biocleaning technologies and their applications. To date, attention has mainly been paid to testing and confirming the effectiveness of bioprocesses and to optimizing procedures and applications. These included investigation into microorganisms specificity, reducing the required treatment time, the number of applications required and the economic costs, as well as the adoption of more efficient delivery systems among others (Antonioli et al., 2005; May et al., 2008; Bosch-Roig et al., 2013b; Troiano et al., 2013). However, little effort has been dedicated to studying possible future developments for bio-restored CH (Lustrato et al., 2012) and, therefore, little is known about their safety. Consequently the use of viable microorganisms to clean cultural heritage has given rise to a number of questions about the risks of these methods and restorers and public and private sector restoration committees are asking the scientific community for more information about the safety of this technology. The questions needing to be answered are: “Is this technology really safe for CH?”, “Is the highest potential gain greater than the highest potential risk?” and “Is this attractive microbiological approach potentially hazardous?”

In our opinion more importance must be paid to the safety of these biocleaning technologies for the Cultural Heritage, the restorers and the environment. Surfaces which have previously been cleaned, which are being cleaned and to be cleaned using biocleaning technologies must be monitored in order to confirm and validate the biocleaning process. More research must therefore be conducted in order to verify that these advanced technologies are really softer and safer, even when the cleaned artwork is subsequently relocated to indoor or outdoor conditions. Short-term and long-term surveillance and monitoring of any developments over time will provide significant information, which would answer questions satisfactorily and represent a guarantee of safety.

Importance must be given to developing suitable strategies for inspection and to monitoring any new microbial interactions on biocleaned artworks. When possible, these should include adequate on-site technologies based on non-invasive tools to understand the potential risks on biocleaned tangible heritage and include physical-chemical, biological, and aesthetic analyses. Similarly, further research and clear demonstrations of the complete safety of biocleaning is of fundamental importance because this technology has a very significant role to play in the introduction and diffusion of a new approach to the application of human-friendly, environmentally-sustainable techniques and technologies for the conservation and restoration of heritage properties. Only through growing awareness of this philosophy will the preservation of the cultural heritage left by our ancestors not occur at the expense of degradation, as is, sometimes sadly happening currently through the use of traditional toxic organic solvents and aggressive techniques and products.

We believe that the confirmation of the absence of risk of these bio-application methodologies will permit their diffusion and application for the removal several different types of alterations on a variety of artistic materials around the world (paints on wood, textiles, paper, papyrus, and so on, as in addition to stone materials and frescoes). The confirmation of the safety of these technologies will also lead to the standardization of protocols, evaluation of end-user costs, and commercialization and, therefore, the production development of innovative “ready to use” products for biorestoration.

## Perspectives

Easily-applied, ready-to-use biocleaning products, that include fast application and removal from altered surfaces, will finally become a reality, when the above-mentioned doubts about safety issues have successfully been clarified. At that time appropriate cost-to-benefit evaluation will confirm biocleaning technology to not only be environmentally sustainable, but also economically viable.

We believe that on the basis of the before mentioned considerations, more European Community projects should finance research on these matters.

### Conflict of interest statement

The authors declare that the research was conducted in the absence of any commercial or financial relationships that could be construed as a potential conflict of interest.
